# The SERTAD protein Taranis plays a role in Polycomb-mediated gene repression

**DOI:** 10.1371/journal.pone.0180026

**Published:** 2017-06-30

**Authors:** Pranabananda Dutta, Willis X. Li

**Affiliations:** 1 Department of Medicine, University of California San Diego, La Jolla, California, United States of America; 2 Department of Biomedical Genetics, University of Rochester Medical Center, Rochester, New York, United States of America; Instituto Gulbenkian de Ciencia, PORTUGAL

## Abstract

The Polycomb group (PcG) proteins have been implicated in epigenetic transcriptional repression in development, stem cell maintenance and in cancer. The chromodomain protein Polycomb (Pc) is a key member of the PcG. Pc binds to the histone mark, trimethylated histone 3 lysine 27 (H3K27me3), to initiate transcriptional repression. How PcG proteins are recruited to target loci is not fully understood. Here we show that the *Drosophila* SERTA domain protein Taranis (Tara) is involved in transcriptional regulation of Pc target genes. Embryos lacking Tara exhibit a partial homeotic transformation of cuticular the segments, a phenotype associated with the loss of *Pc* function. Moreover, *Drosophila* embryos homozygous for a *tara* hypomorphic allele also misexpress *engrailed*, a Pc-regulated gene, and this phenotype is associated with the loss of Pc binding to the *cis* response element in the *engrailed* enhancer. In relation to that, Pc recruitment is reduced on the salivary gland polytene chromosomes and specifically at the *engrailed* locus. These results suggest that Tara might be required for positioning Pc to a subset of its target genes.

## Introduction

Epigenetic mechanisms dictate the developmental fates of cells in an organism. Once differentiation is complete, transcriptional memory is normally retained in every cell across cell divisions. Two important classes of proteins, implicated in this dynamic and multifaceted process, are highly conserved members of the Polycomb (PcG) and Trithorax (TrxG) groups [1]. The PcG proteins are transcriptional repressors whereas the TrxG proteins counteract transcriptional silencing by antagonizing PcG function. The PcG proteins include members such as Enhancer of zeste and Polycomb (Pc). Enhancer of zeste is a SET domain methyl transferase that methylates histone 3 at lysine 27 to create trimethylated histone 3 lysine 27 (H3K27me3), a repressive chromatin mark. Pc is a chromodomain protein and key member of the PcG, which specifically recognizes and binds to the H3K27me3, leading to silencing of its target genes [2].

Polycomb-mediated repression depends on *cis* DNA sequence known as the Polycomb Response Element (PRE) [3]. In *Drosophila*, PREs contain overlapping DNA binding sequences for Zeste, GAGA factor, Dsp1,Pleiohomeotic and ADF1 [4–6]. PcGs also exhibit long-range chromosomal interaction in the nuclear environment, where repressed loci contact each other. The PREs in the *Bithorax* complex (*BX-C*) demonstrate this type of three-dimensional chromosomal interactions [7–9]. During *Drosophila* development, the dynamic interplay between *PcGs* determines segmental identity along the anterio-posterior axis of the developing embryo [10]. Genetic manipulation of *PcGs* results in homeotic transformation in the abdominal segments of the embryo. This is manifested in the misexpression of genes located in the *BX-C* homeotic cluster namely *Ultrabithorax* (*Ubx*), *abdominal*-*A* (*abd*-*A*) and *Abdominal-B* (*Abd-B*) among many other targets [11,12]. Potentially, the PcGs control more than 300 genes as determined by genome-wide chromatin immunoprecipitation [13]. However, the complexity of PcG mediated repression is yet to be fully understood in the context of individual target genes.

**Fig 1:**
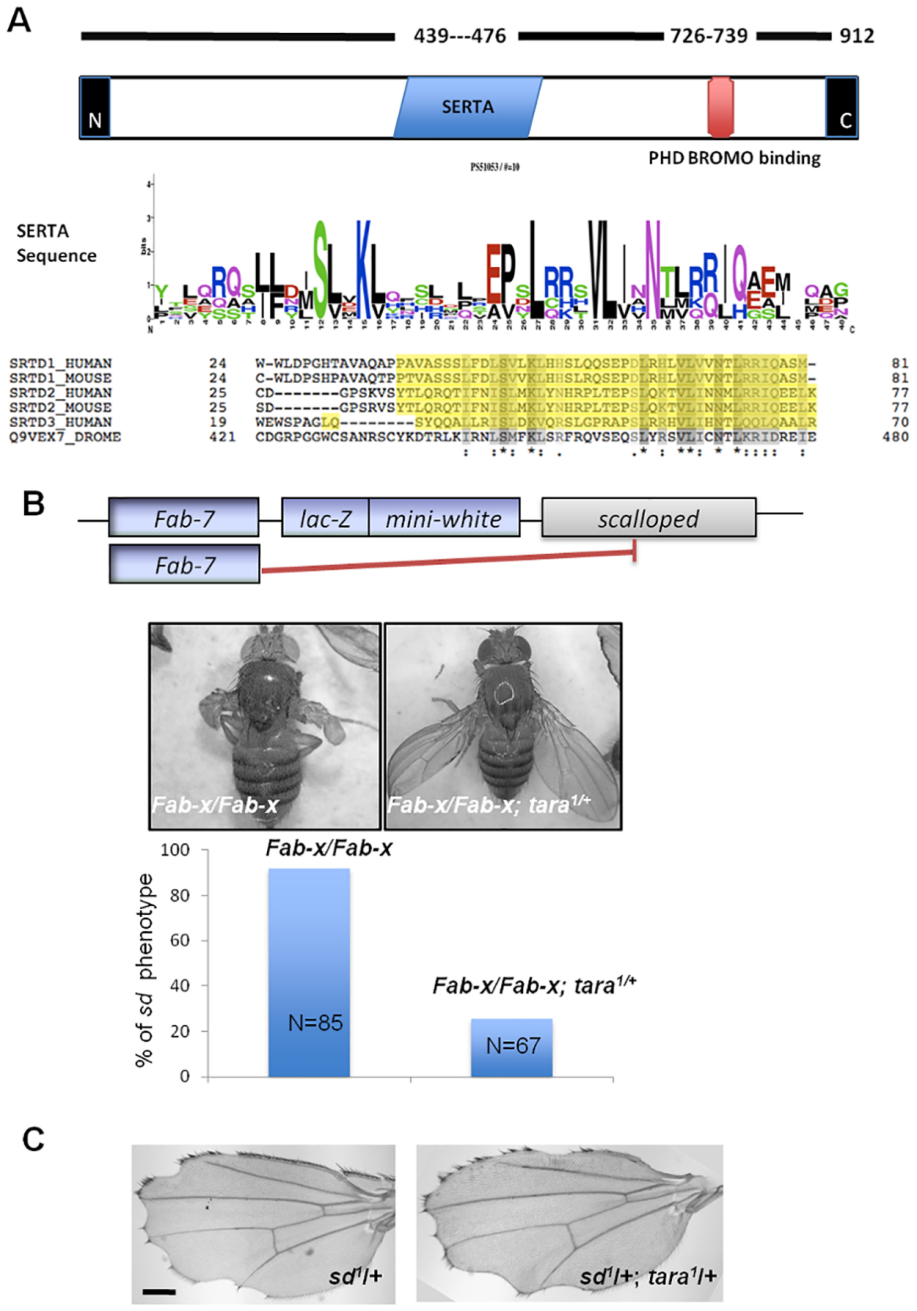
*Taranis* modulates Polycomb mediated gene repression. **(A)** Schematic of the Taranis protein with location of the SERTA and the putative PHD and Bromodomain binding domain (not to scale). The consensus SERTA sequence and its similarity with mouse and human proteins is shown below (**B)** Transgenic PRE mediated pairing sensitive silencing of *scalloped* (*sd*) is suppressed in *tara* loss of function. Simplified representation of the *Fab-X* locus is shown (not to scale). *Fab-X* flies carrying transgenic Fab-7 PRE on the X chromosome show the malformed wing blade phenotype, when raised at 29°C. *tara*^*1*^ allele suppresses this phenotype as shown by the suppression in the *sd* phenotype for both wings. (**C)** *tara* does not genetically interact with *scalloped*. Hypomorphic s*d*^*1*^ allele shows jagged wing margins in males, which is not modified by *tara*.

*Drosophila* SERTA (SEI, RBTA and Tara) protein Taranis (Fig 1A) was identified as a modifier of PcG and trxG [14]. Tara protein shows two separate motifs—Plant Homeodomain (PHD)-bromodomain binding domain (PHBBD) followed by SERTA (SEI, RBTA and Tara) motif arranged from the N-terminal to the C-terminal [14] (Fig 1A). PHD domain proteins can recognize acetylated histone tails, therefore are important modulators of chromatin modifications [15]. The bromodomain is also critical for recognition followed by docking onto acetylated lysine in histone proteins [16]. Hence, The PHBBD in Tara indicates possible important functions in chromatin regulation. The SERTA domain proteins are conserved family of transcriptional factors. However, the function of SERTA proteins in epigenetic regulation is not fully understood.

In this study, we provide genetic and biochemical evidence consistent with a role for Tara in PcG-mediated gene silencing. We find that Tara contributes to Pc recruitment to a subset of loci and modulates PcG target gene expression during *Drosophila* development. Specifically, we show that Tara influences the *engrailed* PRE activity, thereby regulating *engrailed* expression in the developing *Drosophila* embryo.

## Materials & methods

### Fly stocks and crosses

The following stocks are from the Drosophila Stock Center at Bloomington, IN: *tara*^*1*^*/TM3* hypomorphic allele due to P(*lacW*) insertion [14], *tara*^*03881*^*/TM3* P(*Pz*) insertion, hypomorphic *[17], Pc*^*3*^*/TM1*, *Ubx*^*Cbx-1*^,*Ubx*^*1*^, *hsflp;;FRT82B*,*ubi-GFP/TM3*,*Tubulin80-gal4*,*UAS-GFP/CyO*. The *Fab-X* flies were from G. Cavalli (CNRS, France). *Sgs3-Gal4* was from Dirk Bohmann (Rochester, NY). *UAS-tara RNAi* (Transformant ID: 34362) was from Vienna *Drosophila* RNAi Center (VDRC; Vienna, Austria). Salivary gland clones were generated by crossing *hsflp;;FRT82B*,*Ubi-GFP/TM3* (females) with *FRT82B*,*tara*^*03881*^*/TM3* (males).

### Immunostaining of the salivary glands

Embryos from the cross between *hsflp;;FRT82B*,*Ubi-GFP/TM3* and *FRT82B*,*tara*^*03881*^*/TM3* were heat shocked in a 37°C degree water bath for 1hr and left to grow till third instar at 25°C. Salivary gland were dissected in ice-cold PBS and fixed in 4% formaldehyde in PBS for 20 minutes at RT. After washing with 0.3% Triton X 100 in PBS, salivary glands were placed in primary antibody at 4°C overnight. Primary antibodies were diluted in PBS-0.3% Triton x 100, 1% BSA and 1% Normal goat serum. Incubation with secondary antibody was performed at RT for 3hrs and sample mounted with Vectashield DAPI (4’, 6-diamidino-2-phenylindole, Vector Lab, Catalogue# H-1200).

### RNA extraction and real-time qPCR

Total RNA from Drosophila embryo was extracted using Trizol and cDNA was synthesized with Superscripts III. Primers

rp49F: TCC TAC CAG CTT CAA GAT GAC

rp49R: CAC GTT GTG CAC CAG GAA CT

taraF: CAAGCGCGCGTAATTCAAAGT

taraR: AAGCGGCATCACAGAACTT

### Antibodies used for immunostaining

The following antibodies were used: 4D9 mouse anti engrailed/invected (DSHB, 1:10), 1A2E9 mouse anti-Abd-B (DSHB, 1:10), mouse anti Ubx FP3.38 (DSHB, 1:10), rabbit anti-Pc (Santa Cruz, 1:250), sheep anti-DIG (1:50, Roche cat no. 11333089001). All secondary antibodies are from Molecular Probes (Invitrogen): goat anti-mouse or goat anti-rabbit Alexa fluor 488, Alexa Fluor 546, Alexa Fluor 660. All of these were used at 1:250 dilutions. For the sheep anti-DIG primary antibody, a donkey anti-sheep Cy3 antibody was used at a 1:100 dilution.

### Polytene chromosome squash and fluorescent in situ hybridization followed by immunostaining

Salivary glands were dissected from two wandering 3rd instar larvae and fixed for 1 min in a drop of the solution 1 (50ul Triton X-100, 400 μl PBS and 50 μl 37% p-formaldehyde) on a coverslip. Glands were then move to about 40ul of the solution 2 (50ul 37% p-Formaldehyde, 200 μl De-ionized water and 250 μl glacial acetic acid) on another coverslip. After 10mins, the coverslip was taken up on a poly-L-Lysine coated glass slide. The coverslip was tapped with the erasure end of a pencil to break up the salivary glands and spread the chromosomes. The slides were placed in a -80°C freezer for at least 10mins. The coverslip was then removed with a sharp blade and the slides were washed twice in PBS followed by blocking in PBS- 0.3% Triton-X 100 plus 2% BSA (Bovine Serum Albumin Fraction V). For antibody staining, 20 μl of diluted primary antibody was added onto slide area containing the squashed chromosomes (marked with a glass marker) and covered with a 22mmx22mm piece of parafilm followed by overnight incubation at 4°C. The following day, slides were washed twice (15mins each) in PBS-T and incubated at RT for 2 hrs with the secondary antibody. The slides were mounted with Vectashield DAPI.

For FISH, the protocol from Epigenomic NoE (http://epigenesys.eu) was adapted. After polytene squash, the slides were placed in 2xSSC (diluted from 20x stock: 175.3g NaCl, 88.2g Sodium citrate in 1 L deionized water, pH 7.0) for 45mins at 72°C and dehydrated in 70% and 95% ethanol (2x, 5mins each). DNA was denatured with 0.1N NaOH and the slides were washed with 2xSSC for 5mins. The slides were again dehydrated with ethanol and 14 μl probes in hybridization buffer (50% deionized formamide, 0.3M NaCl, 20mM Tris, 5mM EDTA, 1x Denhardt's solution, 10% dextran sulfate, 10mM DTT, 500ug/ml yeast tRNA) was added onto each slide, covered with a glass slide and sealed with rubber-cement. Hybridization was carried out at 37°C for overnight in a moist chamber. After hybridization, slides were washed in 2xSSC and transferred to PBS and immunostaining to detect DIG. FISH was performed with sheep anti-DIG (2hrs RT) and Donkey anti-sheep Cy3 (2hrs RT). Polycomb or anti-flag staining was performed as mentioned before.

The *engrailed* genomic region specific probe was made using the Roche DIG labeling Kit according to manufacturer recommendation. An 8kb Sph1 fragment from *engrailed* locus (P(EN1)) plasmid described in [18]) was used to generate DIG labeled probes. This 8kb *engrailed* genomic DNA also contains the PRE necessary for *engrailed* regulation. We used an 8kb fragment form the engrailed promoter region [18]. This fragment was generated by SPHI restriction digest of P(EN1) plasmid mentioned in 19.

### Chromatin immunoprecipitation from Drosophila larvae

Larvae were grown on apple agar plates at 29°C with excess yeast paste. Approximately 1mg of 2nd instar larvae of the indicated genotypes were crushed in PBS with a pestle. The larval cells were cross-linked at room temperature for 15 minutes with 1% final concentration of formaldehyde, then 0.125mM final concentration of glycine was added and the samples were incubated for another 5 minutes at room temperature. The cells were collected by centrifugation and washed 3 times with PBS-T (1X PBS, pH 7.6 with 0.3% Triton-X). After that, cell lysis buffer was added (50mM HEPES-KOH, 140mM NaCl, 1mM EDTA, pH 8.0, 1% Triton-X, 0.1% Sodium Deoxycholate, 5mM PMSF, 1X PIC) and the cells were sonicated 8 times with 15 second pulses with a Branson S-450 Sonicator set at 40% and output 5. The sample was centrifuged at 13,000xg for 2 minutes at 4°C to remove the cell debris. A portion of chromatin lysate was heated in Elution Buffer (1% SDS, 100mM NaHCO3) at 65°C overnight and input chromatin was prepared using the QIAquick PCR Purification Kit (Qiagen Cat. 28106). The remaining chromatin lysate was incubated overnight in 4°C with 4 μg of anti-Polycomb (Santacruz Biotech, Sc-25762) or an equivalent volume of water for mock treatment. Agarose-G beads were pre-blocked overnight at 4°C in 1.5 μg salmon sperm DNA per 20 μl beads, then 20 μl was added to the chromatin lysate and the samples were kept on a rocking platform overnight at 4°C. The beads were washed three times for 5 minutes each wash in 4°C in Wash Solution (0.1% SDS, 1% Triton-X, 2mM EDTA, 150mM NaCl, 20mM Tris-Cl pH 8.0), then 2 hours at 4°C in Final Wash Solution (0.1% SDS, 1% Triton-X, 2mM EDTA, 500mM NaCl, 20mM Tris-Cl, pH 8.0). The beads were incubated in room temperature for 20 minutes in Elution Buffer and de-cross linked overnight at 65°C. Chromatin was isolated using the QIAquick PCR Purification Kit and 1ul was used for each Sybr Green qPCR reaction. QPCR was performed using standard settings in a Biorad iCycler IQ5 instrument. The following sets of primer sets were used from [19].

*engrailed* PRE forward: 5'-GTTCACTCCCTCTGCGAGTAG-3'

*engrailed* PRE Reverse: 5'-GAAAACGCAGATTGAAACGTC-3'

*engrailed* Gene Forward: 5'-CGCCTTAAGGTGAGATTCAGTT -3'

*engrailed* Gene Reverse: 5'- GGCGGTGTCAATATTTTGGT-3'

### Western blotting

Total lysate was prepared by homogenizing 10 pairs of salivary gland of the indicated genotypes in 20 μl of cell Lysis Buffer and separated on a 10% gel. Pc antibody (Santacruz Biotech, Sc-25762) was used with 1:500 dilutions and anti-rabbit HRP was used as the secondary.

## Results

### Tara modulates a transgenic PRE-mediated phenotype

Tara has been previously implicated in PcG/TrxG function [14,20]. We have formerly identified *tara* in a genetic screen as a potential new component of the RNAi pathway [21]. Since transcriptional silencing mediated by PcGs in certain scenarios requires RNAi components like Argonaute and Dicer [22], we wanted to examine whether Tara could potentially connect PcGs to the RNAi machinery. To this end, we used a transgenic assay system that contains a boundary element for the iab-7 Polycomb response element (PRE) from the *BX-C* locus called *Frontoabdominal* 7 (*Fab-7*) [8,23,24] It regulates selector gene *Abdominal-B* in the wild type context. We used the *Fab-X* line, a specific transgenic insertion on X of a 3.6kb fragment from *Fab-7* locus containing both the boundary and the respective PRE. Fab-x flies harbor two copies in tandem [8,25]. Interestingly, *Fab-X* silences the nearby *scalloped* (*sd*) gene along with the *mini-white* reporter, resulting in a stumpy wing phenotype (referred to as *sd* phenotype) [8] (Fig 1B). The silencing of *sd* occurs only in homozygotes (females in this case) when raised at 29°C, and is dependent on the Polycomb group of proteins and RNAi components like *Dicer* and *Argonaute*, suggesting that the interaction between PcG and RNAi is required for the silencing process [8,22].

We tested whether *tara* can modulate the *Fab-X* induced *sd* wing phenotype. We observed that, the heterozygous *tara* mutant alleles could partially suppress the *sd* phenotype (Fig 1B). The *tara*^*1*^ and *tara*^*03881*^ are two independent alleles both caused by a P-element insertion at the 5’ region of the *tara* locus. Both alleles are associated with embryonic lethality in homozygotes, and they are phenotypically indistinguishable. Expression of *tara* from a transgene under an ubiquitin promoter rescues the lethality, suggesting the lethality is due to loss of *tara* [14,17,20]. In the absence of *tara* mutations, more than 90% of the *Fab-X* flies show *sd* phenotype affecting both wings. In *tara* loss-of-function heterozygous background, we found that this phenotype is reduced to 25%. On the other hand, *tara* heterozygosity does not suppress wing phenotypes, which is caused by a hypomorphic *scalloped* allele *sd*^*1*^ (Fig 1C) [26], suggesting that the observed suppression was unlikely due to genetic interaction between *scalloped* and *tara*. Thus, Tara may play a role in *Fab-X* induced silencing of the nearby *sd* gene.

### Loss of *tara* function leads to homeotic transformation

To understand whether Tara plays a role in Pc function, we investigated the biological function of *tara* during *Drosophila* development. We examined *Drosophila* embryos homozygous for the *tara* mutations. Since both *tara* loss-of-function alleles show similar phenotype in relation to homeotic transformation in the embryos, we focused on *tara*^*03881*^ to investigate Polycomb related mechanisms. Previous RNA in situ hybridization indicates that the maternal component for *tara* is minimal, as the general pattern of its expression is mostly ubiquitous. However, it is not significantly expressed before the developmental stage 5 [14]. Thus, we used zygotic mutant embryos lacking *tara* for this study.

**Fig 2:**
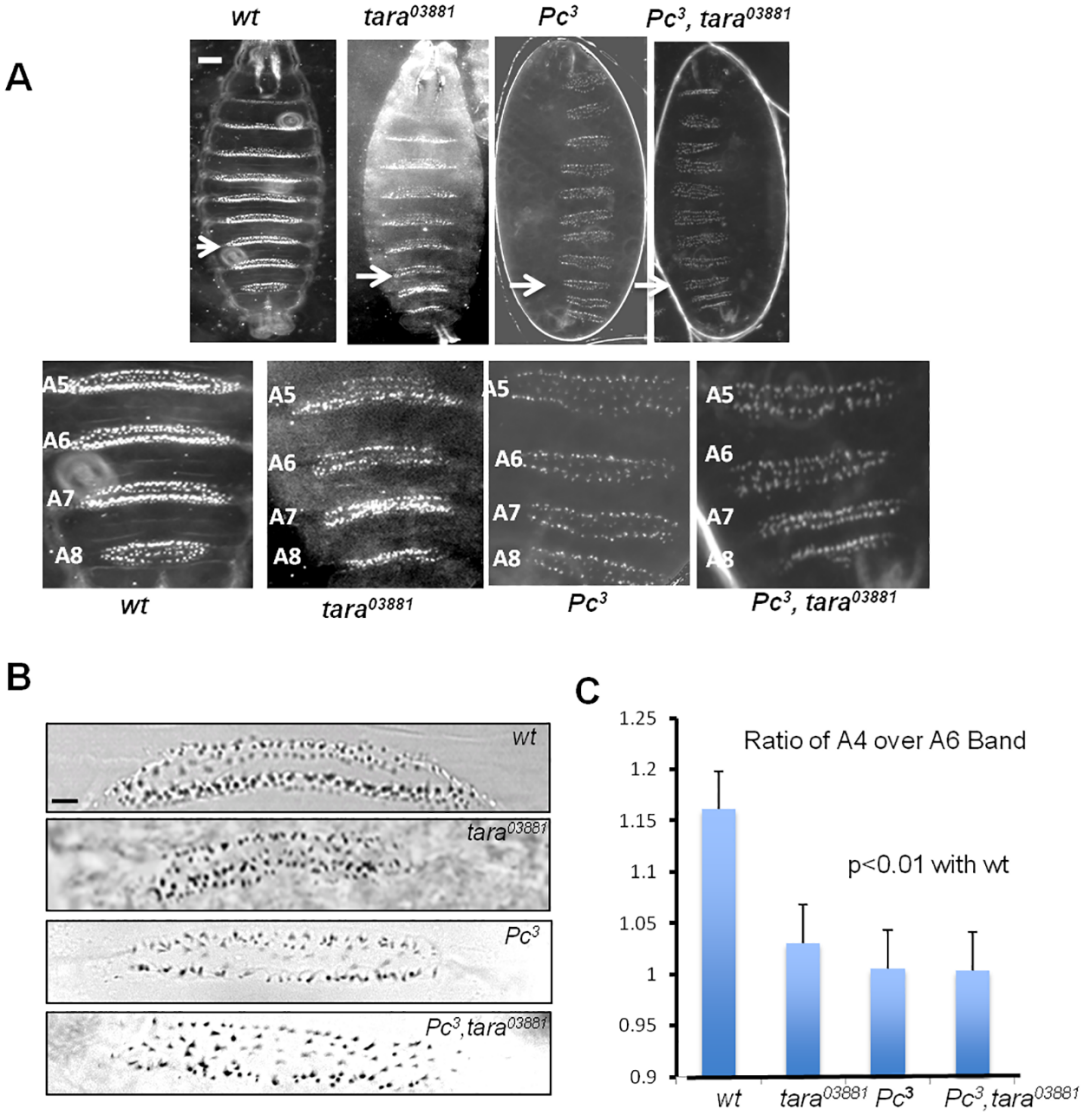
*Taranis* loss-of-function causes partial homeotic transformation. **(A)** Darkfield images of embryos (10x) are shown anterior up. From left to right are *wild type*, homozygous mutants for *tara*^*03881*^, *Pc*^*3*^, and *Pc*^*3*^, *tara*^*03881*^. N = 15 embryos for each genotype were examined, and representative embryos are shown. Magnified view of the A5-A8 denticle bands are shown below for each genotype. Scale bar 20 μm. **(B)** 20x Brightfield illumination of the A6 band from each embryo in A is shown in panel B. Scale bar 10μm. (**C**) The ratio of the denticle band length A4 to A6 for the indicated genotype was show with standard errors (n = 4 for each genotype). p is calculated using Student’s t-Test.

During development, *Drosophila* embryos generate unique patterns of cuticular bristles on the ventral side. These "denticle bands” are arranged in three thoracic (T1-T3) and eight abdominal segments (A1-A8) from anterior to posterior region of the embryo [27] (Fig 2A). Each denticle band has a distinct length and shape and it is thought to be representative of the segment’s identity in the embryo (Fig 2B). In wild-type embryos, the A2-A7 bands in the embryonic cuticle appear trapezoidal, whereas the A8 is rectangular. Cuticles of *tara*^*03881*^ homozygous embryos show homeotic transformation mostly in the posterior segment, such that abdominal segments following the 5th are transformed to resemble the last abdominal segment (A8) (Fig 2B). The A6 band of *tara*^*03881*^ homozygous embryos is shortened and is rectangular similar to A8, instead of trapezoidal as seen in *wildtype* embryos, suggesting a homeotic transformation towards a posterior segmental identity. This transformation is reminiscent of the well-characterized *Polycomb* phenotype [28], albeit less pronounced. For comparison, a null allele of *Polycomb* (*Pc*^*3*^) is shown [29]. *Pc*^*3*^ embryos show complete homeotic transformation across all segments in the embryo. In these embryos, all other abdominal segments transform into segment A8 (Fig 2A). Double homozygous mutant (*Pc*^*3*^, *tara*^*03881*^) embryos lacking both *tara* and *Pc* do not show further enhancement regarding homeotic transformation, suggesting that the effect of *tara* might be mediated by Pc. Since *tara* predominantly causes transformation in the posterior region of the embryo, we quantified the ratio of the length of A4 over A6 bands (Fig 2C). The length here is measured by the distance between two furthest points for each denticle band. Hence, it is the length of the base of trapezoidal or rectangular shape of the specific band measured. In wild-type embryos, the ratio is ~1.16. In *Pc*^*3*^ homozygous embryos, the ratio is ~1. Similar to *Pc* mutants, the observed ratio in the *tara*^*03881*^ homozygotes is significantly smaller (~1.03) than that of *wildtype* embryos. Expectedly, no statistically significant difference was observed between *Pc*^*3*^ and *Pc*^*3*^,*tara*^*03881*^ double mutant embryos (Fig 2C). The partial homeotic transformation indicates that Tara could be involved in patterning process that is regulated by *PcGs*.

### Tara genetically interacts with of the BX-C gene Ultrabithorax

Since homeotic transformation is attributable to misexpression of Pc-regulated homeotic genes like *Ultrabithorax* (*Ubx*), we next asked whether *tara* could modulate *Ubx* dependent phenotype. *Ubx* is a part of the *Drosophila BX-C* cluster of genes, which act in concert to regulate posterior segmental identity in the embryo [30].

**Fig 3:**
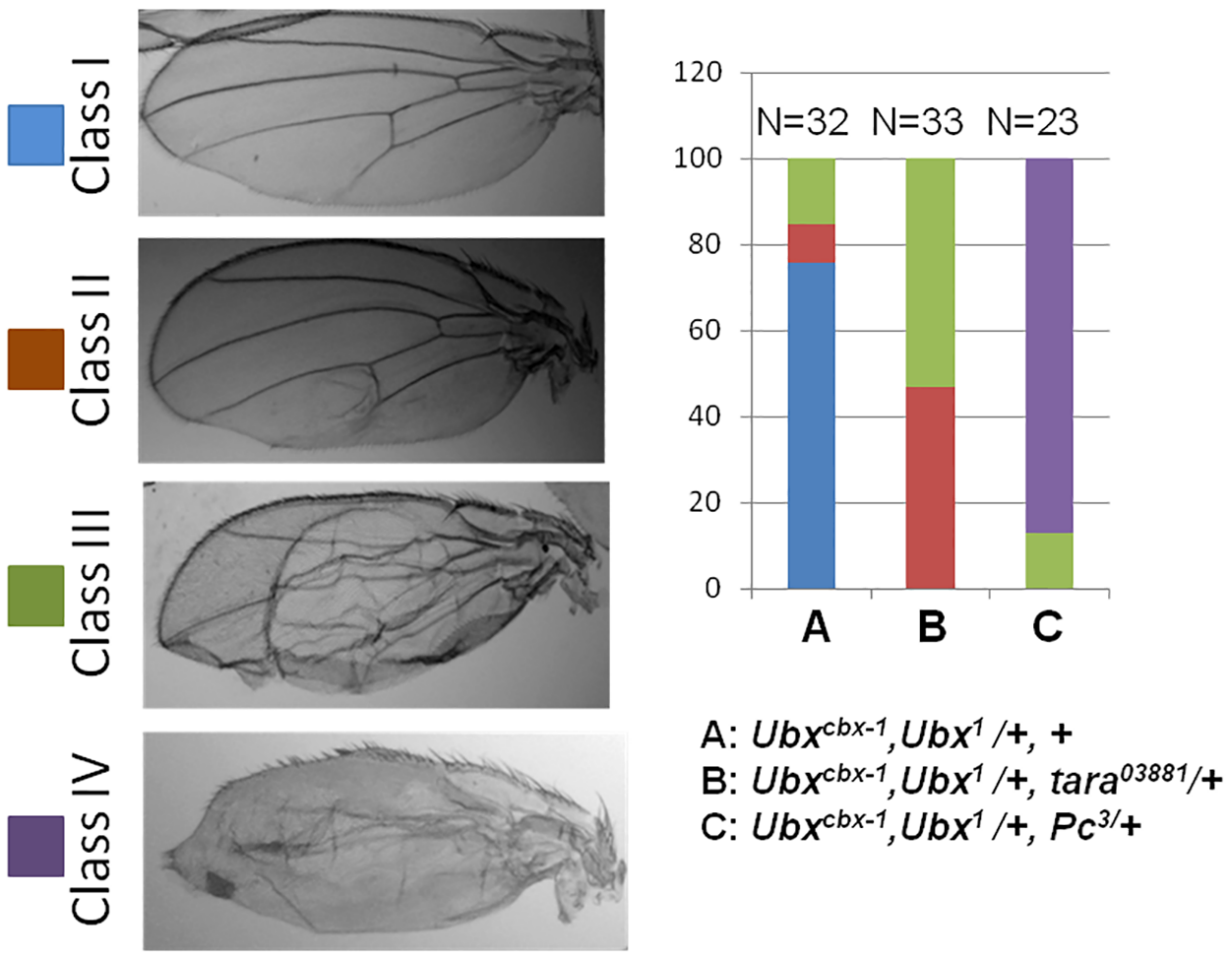
*Tara* interacts with *Ubx*. *tara* enhances *Ubx* dependent wing-to-haltere transformation. The percentage of flies showing the transformation in *Ubx*^*Cbx-1*^, *Ubx*^*1*^/+ (A) is increased in the presence of a heterozygous *tara* loss-of-function allele (B). The wing-to-haltere transformation in the presence of *Pc*^*3*^, a *Polycomb* allele, is shown in (C).

To investigate the role of *tara* in *Ubx* regulation, we utilized a gain-of-function allele of *Ubx* called *Ubx*^*Cbx-1*^ [31]. The *Ubx*^*Cbx-1*^ allele arose due to a transposition event causing a 17-kb fragment from *Ubx* upstream region integrating into a downstream intronic region of the gene, causing upregulation of *Ubx* in parasegment 5 [32,33]. Interestingly, even with a *Ubx* loss-of-function in *cis* (*Ubx*^*1*^ allele), *Ubx*^*Cbx-1*^ was able to activate the *wildtype* counterpart in *trans* when present in heterozygous condition (*Ubx*^*cbx-1*^, *Ubx*^*1*^/+), causing ectopic expression of *Ubx* in wing disc, a phenomenon known as transvection [34]. The *Ubx*^*Cbx-1*^, *Ubx*^*1*^/+ heterozygous flies exhibit partial wing-to-haltere transformation, which is typically enhanced in the *PcG* mutant background (Fig 3). This phenotype is fully penetrant but varies in expressivity. We categorized the phenotype into four different classes—I to IV—depending on the severity of the transformation (Fig 3). The Class I category wings has a slight curved region on the posterior boundary of the wing, Class II wings show partial loss of the fifth wing-vein and the area between the 4th and 5th vein appear crumpled. The wing morphology is severely affected in Class III, with the presence of bubbles and crumpled posterior margin. The Class IV wings are the most affected as the wing-to-haltere transformation is prominent in the stumpy wing morphology. The severity of wing phenotype is positively correlated with the expression of *Ubx* in the larval wing disc tissue [32]. At 22°C, about 75% of Ubx^*Cbx-1*^, *Ubx*^*1*^ heterozygous flies show mild transformation (Class I), and only 9% shows Class II and 15% shows Class III phenotypes, respectively. In the presence of one copy of *tara*^*03881*^, the majority of the wings fall into the Class II (46%) and Class III (53%) categories, suggesting that loss of *tara* enhances the wing-to-haltere transformation of Ubx^*Cbx-1*^, *Ubx*^*1*^ heterozygous flies. The wing phenotype, however, is not as severe as that of Ubx^*Cbx-1*^, *Ubx*^*1*^/*Pc*^*3*^ flies, in which 86% of the wings are in Class IV category. These results suggest that Tara normally negatively regulates *Ubx* expression, although Pc appears more important in repressing *Ubx*.

### Loss of *tara* function reduces Pc recruitment to PREs

**Fig 4:**
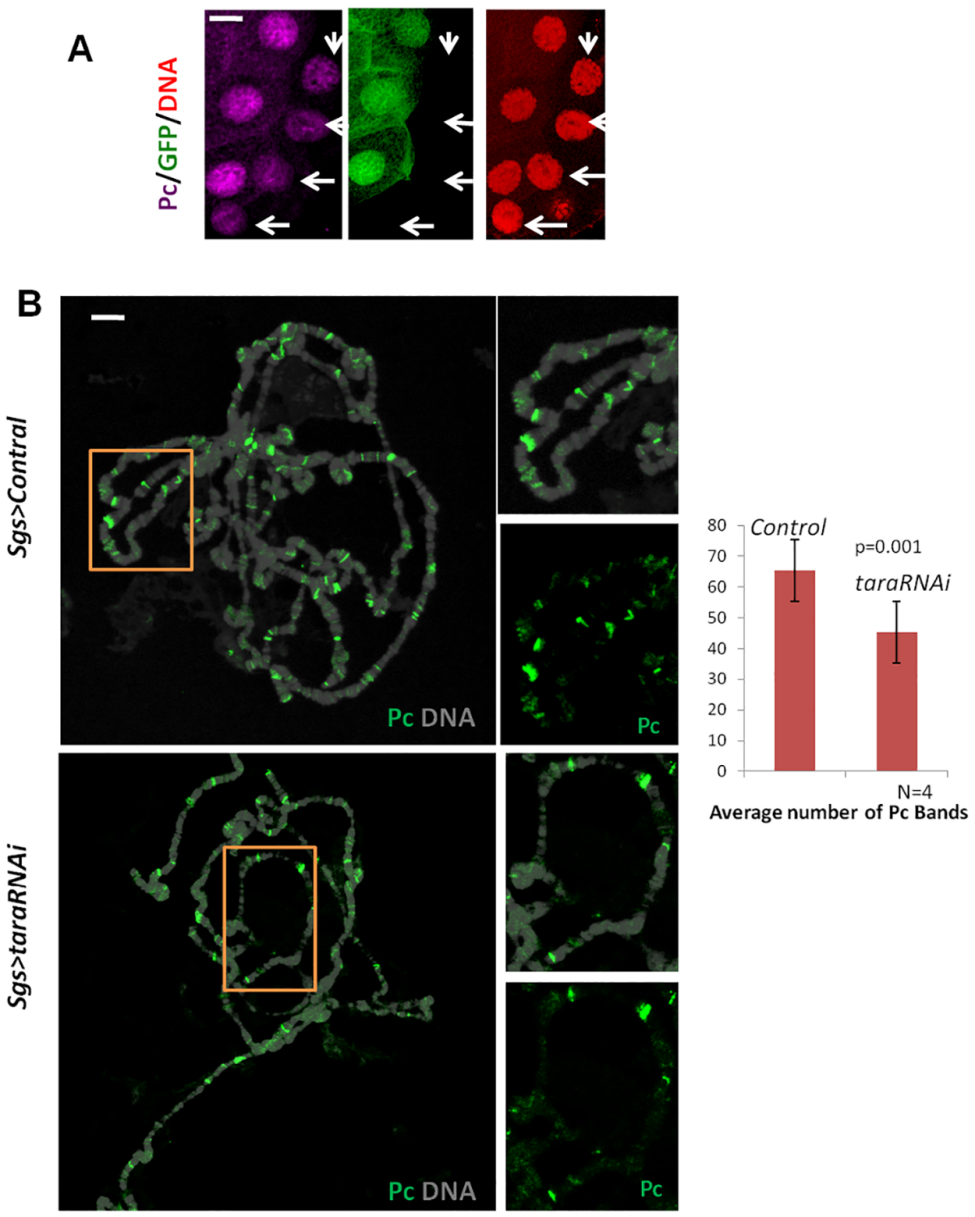
*Taranis* knockdown results in loss of Pc recruitment. **(A)** Mitotic clones in the Salivary gland were induced by heatshock at the embryonic stage and heat-shocked embryos were left to grow until 3^rd^ Instar. Salivary glands from wandering 3rd instar larvae were dissected and immunostained for Pc. The GFP negative cells are *tara*^*03881*^ homozygous clones. Note that *tara*^*03881*^ homozygous cells (white arrow, GFP negative) show less Pc in salivary glands nuclei. GFP positive cells are wild type or heterozygous for *tara*^*03881*^. Genotype of the larvae is *hsflp;;FRT82B*,*Ubi-GFP/FRT82B*,*tara*^*03881*^. (**B)** Salivary glands from *Sgs3 > taraRNAi* or control wandering 3rd instar larvae were squashed and subjected to immunostaining with antibody against Pc to visualize Pc distribution on polytene chromosomes. DNA (DAPI) staining of the chromosome is shown is grey in the merged images. Magnified view of the boxed area on the right indicating reduction in number of Pc bands. Graph depicting average number of Pc bands on polytene chromosome on *taraRNAi* as well as driver only (*Sgs3 >w1118*) control.

The genetic experiments described above indicate that Tara might be involved in Pc function. The suppression of the *Fab-X* and *Ubx*^*Cbx-1*^ phenotypes by *tara* mutations could have resulted from a failure of Pc recruitment to the PRE in *Fab-X* or Ubx regulatory regions. Two of the important steps in the canonical PcG functions are trimethylation of histone 3 lysine 27 (H3K27me3) followed by Pc binding to this epigenetic mark via its chromodomain [35]. Tara could conceivably be involved in either of these processes. We next set out to investigate these possibilities. To this end, we first created mitotic clones using the FLP-FRT technique and examined Pc levels in hypomorphic homozygous *tara*^*03881*^ cells of the 3rd instar larval salivary glands (see Methods). We observed a significant reduction in Pc fluorescence in *tara*^*03881*^ / *tara*^*03881*^ cells (Fig 4A, arrows).

*Drosophila* polytene chromosome shows distinctive pattern of Pc binding as multiple high and low intensity bands. This provides us with the necessary setup to examine Pc binding to chromosomes at higher resolution compared to whole-mount immunostaining in salivary gland. We knocked down *tara* in the salivary gland by expressing dsRNA against *tara* using the UAS-Gal4 system followed by immunostaining with anti-Pc and anti-H3K27me3 (see Methods; S1 Fig). The salivary gland-specific *Sgs3-gal4* driver was used for this purpose to drive RNAi specifically in the salivary gland. We observed a significant reduction in the number of Pc bands in *tara* knockdown (Fig 4B bottom panel) compared with control (Fig 4B top panel), corroborating the observation in *tara* loss-of-function clones. Pc bands were decreased to an average of 44 per nuclei. In comparison, *Sgs3 > w1118* (control) shows 63 Pc bands (Fig 4B, graph). Magnified view of the third chromosome is shown in the panel next to the whole chromosome for better comparison. However, *tara RNAi* appears to decrease the level of Pc in most of the low intensity bands, whereas the number of high intensity bands remains unchanged. Our result indicates that Tara might be important for Pc recruitment at specific target loci but may not be necessary for all the genes regulated by Pc.

We did not observe any significant changes in the levels and distribution of H3K27me3 in polytene chromosomes after knocking down *tara* (S2 Fig). When compared to the *Tubulin80-Gal4* driver only control, there was no significant change in Pc protein levels for embryos with *tara* knockdown (S3 Fig). Our data indicate that Tara might be involved in Pc recruitment rather than affecting histone methylation of H3K27 or the production of Pc per se.

### Misregulation of *engrailed* in *tara* loss-of-function embryo

**Fig 5:**
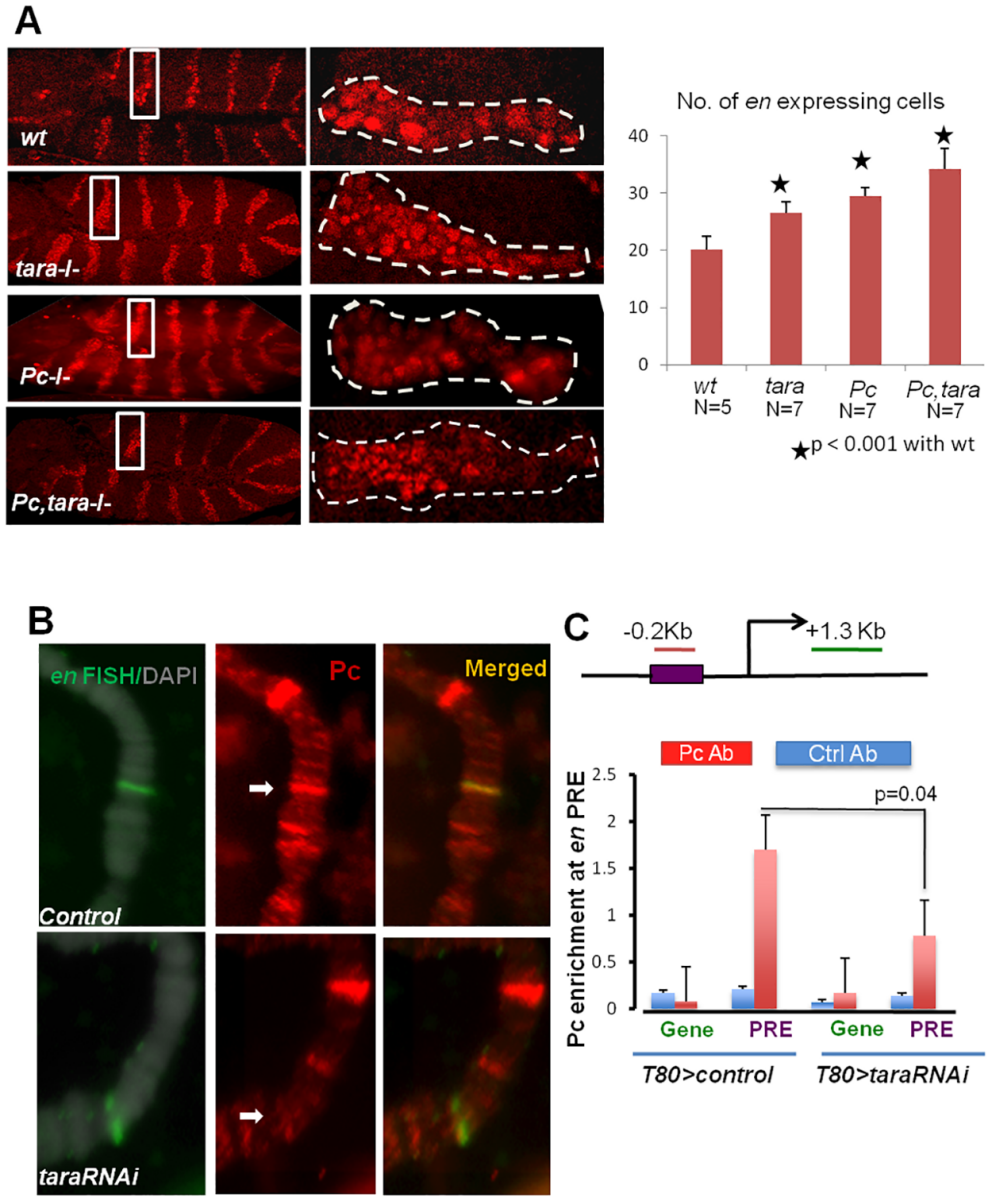
Polycomb target *engrailed* is derepressed in *taranis* loss of function. **(A)** Embryos showing Engrailed immunostaining. The white-boxed area is shown on the right panel with higher magnification for the corresponding embryo. No. of cells was counted in the boxed regions and plotted for comparison with *wildtype*. From top to bottom: *wild type*, *tara*^*03881*^, *Pc*^*3*^ *and Pc*^*3*^,*tara*^*03881*^. Scale bar 10 μm left panel 2.5μm right panel, N = 6 (wt, tara) and 8(pc, pc-tara) **(B)** Salivary glands from *Sgs3 > taraRNAi* or control wandering 3rd instar larvae were squashed and subjected to FISH for the en locus and followed by immunostaining with antibody against Pc. Top: *Sgs3 >w1118*; Bottom: *Sgs3 > taraRNAi* at 29°C. To maximize knockdown, larvae were raised at 29°C. (**C)** *Tubulin80-gal4>UAS-taraRNAi* and control 2nd instar larvae were collected for chromatin extraction. Chromatin IP was performed with primers to amplify an *en* PRE fragment located at 0.2 Kb upstream from the transcription start site and an intergenic fragment located 1.3 Kb downstream. The diagram shows approximate location of the PRE and intergenic region as a negative control (not to scale).

As mentioned previously, Pc function in *Drosophila* is mediated by cis DNA element called PRE. We have found *tara* to be important in the transgene *Fab-X* mediated silencing and possible repression of *Ubx*, both of which require PRE activity (Figs 1B and 3). We wanted to test whether *tara* can affect the PREs of other known Pc targets. *Drosophila* segment polarity gene *engrailed* (*en*) is a Pc target with two well-characterized PREs in a 2.4kb region upstream of its transcription start site. The proximal PRE is located 181bp upstream of the transcription start site and is characterized by Pc binding [36]. We first looked at *en* expression pattern in *tara*^*03881*^ homozygous embryos. *en* is expressed in 14 distinct stripes during embryonic development at germ-band extension [37,38]. In each stripe, *en* expression is limited to 2–3 rows of cells in *wildtype* embryos (Fig 5A). *Pc*^*3*^ homozygous embryos show a misexpression of *en*, as the total number of cells expressing *en* in a stripe increases. We observed a very similar phenotype in the *tara* embryos homozygous for *tara*^*03881*^ (Fig 5A). Interestingly, unlike the homeotic transformation phenotype (Fig 2A), the *tara*^*03881*^ and *Pc*^3^ double mutant embryos show a further increase in the number of cells that express *en* than that of single mutant embryos (Fig 5A). The effect is most discernible in the 13^th^ parasegment. Similar synergistic relationship is known to exist between PcG proteins in the context of *engrailed* regulation [39]. To quantify *en* expression, we counted the number of *en*-expressing cells in the 13th parasegment of each embryo. In wildtype embryos, the *en* stripe in the 13th parasegment contains average 17 cells (Fig 5A, white box and right), whereas in *tara*^*03881*^ homozygous embryo, this number is increased to 22. *Pc*^*3*^ homozygous mutation results in expression of *en* in average 29 cells, and the *tara*^*03881*^ *Pc*^*3*^ double mutant shows enhancement as the number of *en* expressing cells reaches 33 (Fig 5A, white box and right).

### Tara is required for efficient Pc binding to the engrailed PRE

Next, we asked whether misexpression of *en* could be attributable to loss of Pc binding to the *en* locus and PRE. We employed chromatin immunoprecipitation (ChIP) to investigate Pc binding to the *en* locus. For FISH-I we used the *Sgs3-gal4* driver to knockdown *tara* in the salivary gland and examined polytene chromosomes from salivary glands of wandering third instar larvae. Squashed polytene chromosome from control (*Sgs3 >w1118* and *Sgs3 > taraRNAi* were subjected to FISH-I (see Methods). In the driver-only control, a distinct Pc band (red) is coincident with the FISH signal (green) from the *engrailed* locus (Fig 5B, top panel). The intensity of this band is lower than a nearby high intensity Pc band. Knocking down *tara* resulted in a much-reduced Pc signal at the *en* locus (Fig 5B, bottom panel). The nearby high intensity Pc band, however, does not diminish, consistent with our observation that not all Pc bands are affected by knocking down *tara* (Fig 4B). This suggests that Tara might be required for Pc binding to a subset of Pc regulated loci including *en*.

We also examined whether Tara is required for Pc binding to *en* PRE with Chromatin IP (ChIP). We used the ubiquitous *Tubulin80-gal4* driver to globally knockdown *tara*. *Tubulin80 > taraRNAi* and control 2nd instar larvae were collected for chromatin extraction. ChIP was performed with primers described in [19]. One primer set was used to amplify the *en* PRE, located 0.2kb upstream from the transcription start site. The other set of primers were for a fragment located 1.3 kb downstream site in the *en* gene region (Fig 5C), which does not contain any PRE or Pc binding sequence, hence can be considered as an internal negative control. In *Tubulin80 >w1118* larvae, Pc binding to *en* PRE was observed but not in the control *en* gene region (Fig 5C). In contrast, *Tubulin80 > taraRNAi* larvae show reduced Pc binding at *en* PRE (p = 0.04). As expected, the internal gene control did not show any Pc enrichment. These results suggest that Tara is required for the recruitment of Pc to the endogenous *en* PRE to modulate its transcription.

## Discussion

We have investigated the role of Tara in Pc-mediated gene repression in *Drosophila* and have found that Tara modulates the epigenetic silencing of the developmental gene, engrailed, that is transcriptionally regulated by the Polycomb group proteins. The segment polarity gene *engrailed* is a target of the Polycomb-mediated transcriptional repression. We have further shown that Tara is required for Pc binding at of the *engrailed* Polycomb response elements upstream of the transcription start site, although the detailed mechanism awaits further investigation. We have found that Tara genetically interacts with the *Hox* gene *Ubx*. During development, the *Ubx* expression pattern at stage 16 ventral nerve cord seems to be uniform in *tara* loss-of-function embryos, instead of a gradient pattern as found in *wildtype* (not shown). However, this observation does not satisfactorily account for the embryonic homeotic transformation in *tara* loss-of-function. In the developing *wildtype* embryo, *Abd-B* is expressed in represses PS13 and PS14, whereas *Ubx* is absent in those segments. The mis-expression of *Ubx* in Parasegment 13 or 14 was not clearly visible in the *tara*-/- embryos. Alternatively, *tara* loss-of-function embryos did not show any significant change in the *Abd-B* expression pattern either (S4 Fig for *Abd-B*). In PS10-12, *Abd-B* acts in concert with *abd-A* to determine parasegment identity by repressing *Ubx* [11]. However, we have not examined *abd-A* expression in our study to test the possibility of its deregulation in *tara-/-* embryos. Since the interplay between these *Hox* genes determines parasegment identities in the wildtype embryo, we are unable to determine the mechanistic roles *tara* might play in this respect. It is possible that, Tara might be indirectly involved in repressing *Hox* genes in cooperation with Pc. Alternatively, Tara could be regulating interacting partners of the Hox transcription factors, which might be required for their activity. Future experimentation will be necessary to tease out the role for *tara* in *Hox* gene regulation. It will be interesting to find how *tara* expression correlates with *Abd-B* and *Ubx*. It is interesting to note that *tara* is expressed at all stages during embryonic development as determined by in situ hybridization [14]. After gastrulation, *tara* expression covers the entire germband of the developing embryo. The expression at stage 11–12, is very strong and broadly covers the embryo.

The regulation of *en* expression is under the control of complex inter-regulation in *Drosophila* embryo and imaginal disc involving PcG and Trx proteins[39–42]. It has been shown that Pc is required for maintaining *en* expression in the posterior par segments in the developing embryo, and in the posterior compartments of imaginal discs [39]. In *tara* loss-of-function mutant embryos, we found that the number of cells expressing *en* increases (Fig 4A), which suggests that more cells adopt the posterior identity. Despite this, the anterio-posterior compartment boundary is still maintained. The En protein is repressed in the anterior region of the wing disc by *PcG* proteins [39]. Surprisingly, the *tara* (*tara*^*03881*^) clones in the wing disc do not show any significant alteration in the *engrailed* expression as revealed by antibody staining (not shown; also see [20]. This might be due to the redundant functions of members of the PcG in repressing *en* [43,44], or that Tara is only required in tissue regeneration after damage [20,45]. In our study, we did not observe any physical interaction between Tara and Pc by co-immunoprecipitation. We also found that Tara localization on polytene chromosomes showed only partial colocalization with Pc band (not shown). We were unable to observe Tara recruitment on the *en* locus, as Tara signal on the polytene chromosome decreases significantly following immune-FISH procedure (not shown). It is likely that Tara might regulate Pc recruitment via indirect mechanisms, which remains to be investigated.

Tara was initially characterized as an enhancer of *trithorax* based on genetic interactions *[14]* and was recently shown to mediate the function of the PcG protein polyhomeotic in repressing *engrailed* expression [20]. Our work provides additional support for the biological functions of Tara as a mediator of PcG functions. It is noted that similar dual functions have been observed with other member of the Polycomb and Trithorax family [46], which include the histone methyltransferase Enhancer of zeste (E(z)), a trithorax mimic of *E(z)*, *called E(z)*^*trm*^ [47,48], *Additional sex combs* (*Asx*) [49], *Corto* [50], and *batman* [51]. Thus, PcG/trx dependent transcriptional regulation is complex and is context-dependent. Tara could be part of this multifaceted network and might behave as a PcG member for repression of certain targets while as a Trithorax member for activation of certain other genes. However, genes that are positively regulated by Tara have not yet been identified. It will be interesting to explore interaction between effectors of context-dependent transcriptional regulators and Tara in the future.

## Supporting information

S1 Fig Levels of Taranis mRNA after knockdown. Total RNA was isolated from 1^st^ instar larvae in which *tara RNAi* was expressed with the ubiquitous *Tubulin80-gal4* driver. Driver only control is shown on the right. *rp49* mRNA was used as control for total mRNA levels.
(PDF)

S2 Fig Tara does not modulate histone 3 lysine 27 trimethylation (H3K27me3). RNAi construct for tara or control was expressing using the salivary gland driver Sgs3 and the 3^rd^ instar salivary gland polytene chromosomes were immunostained with anti-H3K27me3 (left) or with DAPI. No differences in H3K27me3 level or pattern were found between the two samples.
(PDF)

S3 Fig Tara does not regulate Pc protein levels. Lysates from 10 pairs of salivary gland from control and *tara* knockdown conditions were subject to Western blotting with anti-Pc and anti-H3 (loading control). Note that knocking down *tara* did not affect Pc protein levels.
(PDF)

S4 Fig Abd-B expression in *tara* mutant embryos. Wild-type and *tara*^*03881*^ homozygous embryos were subject to whole-mount immunofluorescence with anti-Abd-B antibody.
(PDF)

## Abbreviations

ChIP: Chromatin Immunoprecipitation
FISH: Fluorescent in situ hybridization
Pc: Polycomb
PcG: Polycomb Group
PRE: Polycomb Response Element
