# Supplementary figures and images for: The SERTAD protein Taranis plays a role in Polycomb-mediated gene repression
Source: PLoS One. 2017 Jun 30;12(6):e0180026. doi: 10.1371/journal.pone.0180026 (PMC5493352; doi:10.1371/journal.pone.0180026)

S1 Fig

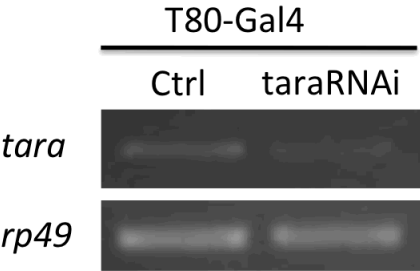

Supplement: S1 Fig — Total RNA was isolated from 1st instar larvae in which tara RNAi was expressed with the ubiquitous Tubulin80-gal4 driver. Driver only control is shown on the right. rp49 mRNA was used as control for total mRNA levels. (PDF) [file pone.0180026.s001.pdf]

S2 Fig

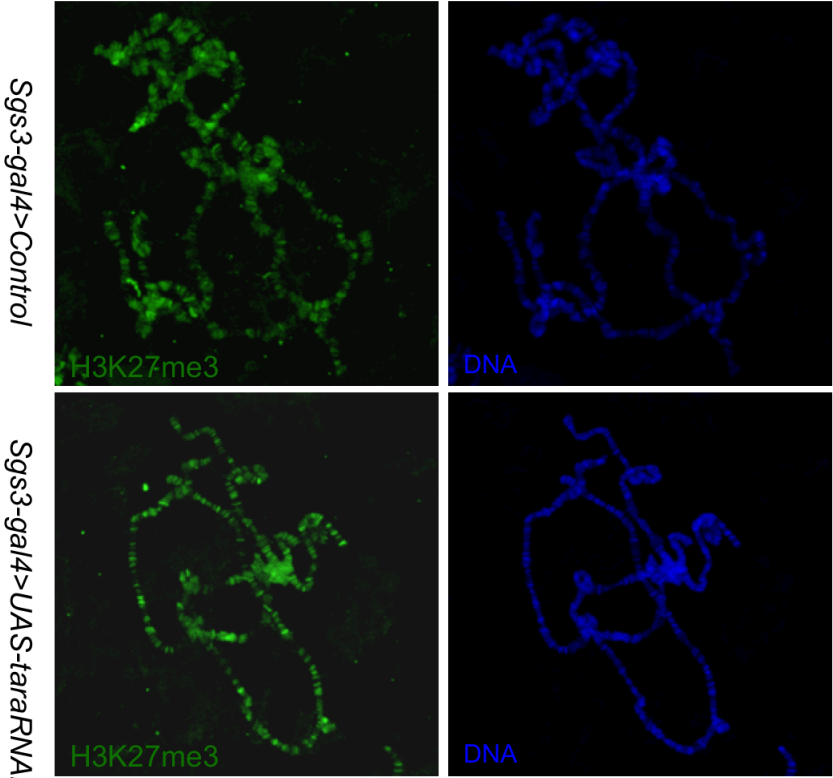

Supplement: S2 Fig — RNAi construct for tara or control was expressing using the salivary gland driver Sgs3 and the 3rd instar salivary gland polytene chromosomes were immunostained with anti-H3K27me3 (left) or with DAPI. No differences in H3K27me3 level or pattern were found between the two samples. (PDF) [file pone.0180026.s002.pdf]

S3 Fig

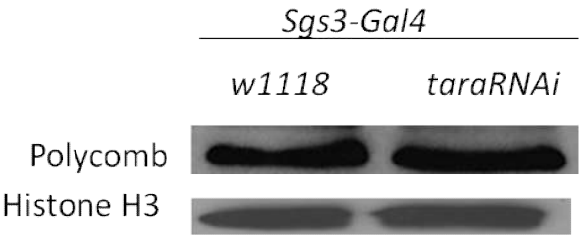

Supplement: S3 Fig — Lysates from 10 pairs of salivary gland from control and tara knockdown conditions were subject to Western blotting with anti-Pc and anti-H3 (loading control). Note that knocking down tara did not affect Pc protein levels. (PDF) [file pone.0180026.s003.pdf]

S4 Fig

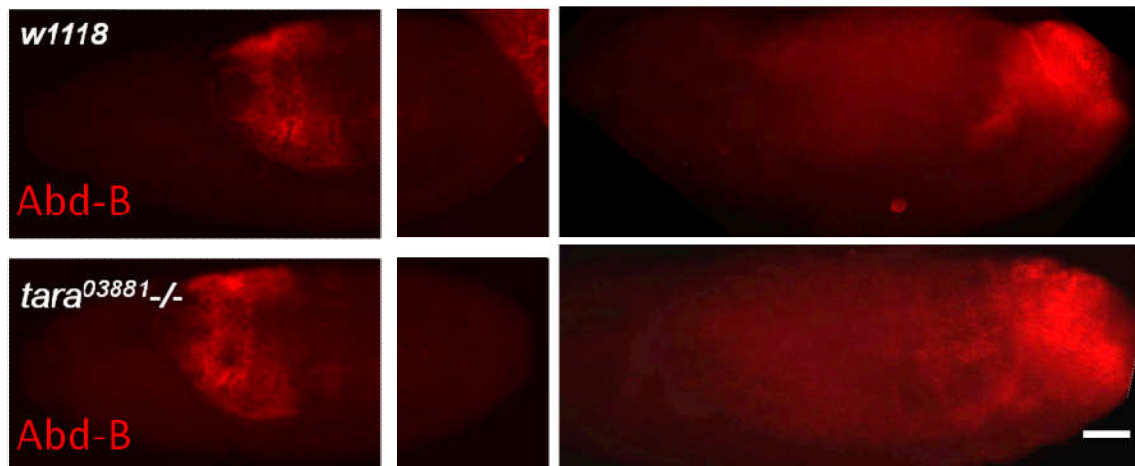

Supplement: S4 Fig — Wild-type and tara03881 homozygous embryos were subject to whole-mount immunofluorescence with anti-Abd-B antibody. (PDF) [file pone.0180026.s004.pdf]
